# Prevalence of diabetic retinopathy in patients with diabetes in Alexandria and North-West Delta, Egypt

**DOI:** 10.1007/s10792-023-02692-4

**Published:** 2023-03-24

**Authors:** Ahmed Elmassry, Islam S. H. Ahmed, Noha Adly, Marwan Torki

**Affiliations:** 1 Ophthalmology Department, Faculty of Medicine, Alexandria University, Alexandria, Egypt; 2 Computer Systems and Engineering Department, Faculty of Engineering, Alexandria University, Alexandria, Egypt; 3 Consultant in the Applied Innovation Center, Ministry of Communications and Information Technology, Alexandria, Egypt

**Keywords:** Egypt, Middle east, Diabetes mellitus, Epidemiology, Glycosylated hemoglobin, Diabetic retinopathy

## Abstract

**Purpose:**

The purpose of this research was to estimate the prevalence of DR in Alexandria and the North-West Delta region.

**Methods:**

All diabetic patients attending the general ophthalmology clinics (Group 1), diabetic internal medicine clinics (Group 2), or reached out in the local communities (Group 3) were eligible to participate. Fundus photographs were graded according to the Scottish DR grading system by three independent UK-certified graders. Adjudication by a consultant was done when needed.

**Results:**

Out of 11,033 screened patients, 10,811 had a gradable fundus photograph in at least one eye and were included. The numbers of cases in groups 1, 2 and 3 were 3940, 2826, and 4045, respectively. Males represented 38.35% of the cases. Mean age was 55 ± 12.63. For the whole sample, groups 1, 2 and 3, the DR prevalence was 32.49, 46.4%, 29.13%, and 21.29%, respectively. The prevalence of proliferative DR (grade R4) was 6.16%, 11.83%, 5.02%, and 1.45%, respectively, and of referable maculopathy (Grade M2) was 19.95%, 31.42%, 15.92%, and 11.59%, respectively.

In univariate analysis, older age, higher random blood glucose, and longer DM duration were associated with a higher risk of both DR and referable diabetic maculopathy. This association was maintained in multivariate analysis for the high random blood glucose level and the longer duration of DM (but not for the older age).

**Conclusion:**

A significantly higher prevalence of DR, grades R4 and M2 was found in the hospital-recruited patients than in diabetics from the local communities.

## Graphical abstract

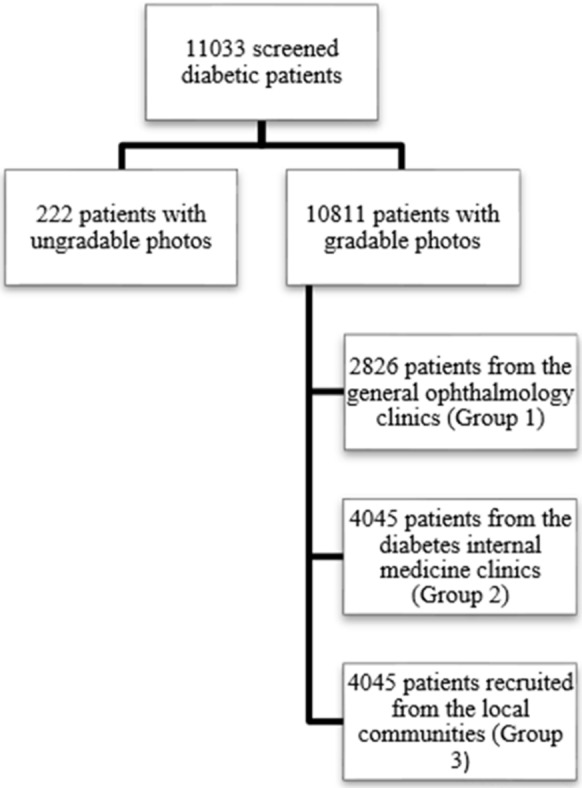

## Introduction

In Egypt, the estimated prevalence of diabetes mellitus (DM) is expected to rise from 5–10% in the 1990s to > 13% of the population over 20 years old by 2025 [1–3].

The estimation of the prevalence of diabetic retinopathy (DR) is challenging due to the great variation in the study populations, methodologies, and grading schemes [4].

Globally, the prevalence of DR and diabetic macular edema (DMO) is expected to rise from the estimates for the period 2015–2019 (27%) due to the expected increase in the life expectancy of people living with DM [4, 5].

There is a paucity of information and a substantial heterogeneity in the DR estimated prevalence in Egypt due to variation in the methodologies and the study populations. Herman et al*.* reported a DR prevalence of 42% in a population-based study that included diabetic patients in Cairo and surrounding rural villages. In another hospital-based study published in 2011, Macky et al. reported that 20.5% of patients with diabetes had DR [6].

In addition, there is a great variation in the reported estimated prevalence of DR in Middle Eastern countries, including Egypt (7.6–60%) [6–15].

The current study aimed at estimating the prevalence of diabetic retinopathy in diabetic patients in Alexandria (the city with the second-largest population after the capital) and the nearby governorates in the north-west Delta. It included three groups of patients: patients attending the general ophthalmology clinics (Group 1), patients attending the diabetic internal medicine clinics in the hospital (Group 2), and those reached in the local community (Group 3).

## Materials and methods

### Study design

We conducted a population-based cross-sectional epidemiological study conducted from March 2021 to January 2022 on patients diagnosed with DM using six mobile screening teams which covered the Governorates of Alexandria, Beheira and Kafr El Sheikh. Participants were enrolled from both the hospital (diabetic patients attending the ophthalmology outpatient clinic or the diabetes internal medicine clinics in the Alexandria Main University Hospital) and the local communities (by campaigns in both the rural and the urban areas).

The research protocol was reviewed and approved by the Ethics Committee of the Faculty of Medicine, Alexandria University. The current study adhered to and was conducted in accordance with the ethical principles of the Helsinki Declaration.

All the included patients signed a written informed consent to participate in this study. Patients in the hospital must have already been diagnosed with diabetes. On the other hand, community participants were recruited by advertisements and were included if they had previously been diagnosed with DM by a qualified physician,

The importance of screening was explained, and screening procedures were provided for free to all the participants.

Each screening team included a social worker who oversaw the recruitment of the patients. Hospital patients were contacted during their visits to the general ophthalmology or diabetic outpatient clinics in Alexandria Main University Hospital. Community patients were recruited by public awareness campaigns using local advertising by way of banners and brochures within the social clubs, markets, factories, mosques, and churches. Upon arriving at the screening site, a trained nurse would collect the personal data of the patient as per his/her national ID card (name, gender, date of birth, address, national ID number which was used to avoid reentry on consecutive visits), the contact phone number, past medical history including the data about DM (type, treatment given, duration defined as the time since the first diagnosis of DM by health personnel, level of glycosylated hemoglobin HbA1c if available).

As we do not have a registry of the diabetic patients in Egypt, we considered the patient to suffer type 1 DM if he/she has never had oral hypoglycemics to control the diabetes, i.e., he/she had insulin prescribed for controlling the DM from the onset of diagnosis. Alternatively, we would consider the patient to suffer type 2 DM if he/she had any history of treatment by oral hypoglycemics. A finger-prick random blood glucose (RBG) test was done by a digital glucometer. A data entry clerk would enter the data into a software program on a provided laptop.

Afterward, color, digital, non-stereoscopic fundus retinal photographs were taken by trained fundus photographers using a nonmydriatic, hand-held auto fundus camera (Optomed Aurora, Optomed, Finland). A single 50° photo centred on the macula was taken for each eye.

The fundus photographs on each camera would be transferred to a cloud storage on daily basis. The software system would anonymize the fundus photographs and make them accessible on a website to the graders.

### Grading

Each fundus photograph was graded by three independent graders. The grading team included at any point in time 12 ophthalmologists with at least 3 years of experience. The online grading software allowed improvement of the image quality by automatic adjustment of the contrast, brightness and magnification to facilitate the evaluation of the details of the fundal photograph.

The graders were able to assess the photographs through a website on their computers. Each photograph was classified as gradable or not. The photograph was considered non-gradable if more than one-third of the photograph could not be assessed or if the field, exposure or focus of the photograph were of poor-quality preventing proper grading.

The Scottish DR grading system (Table 1) was followed to grade the fundal photographs. In the case of agreement of the three graders, their grade would be considered the final grade. In the case of non-agreement, the photograph would be adjudicated by one of two consultant ophthalmologists with at least 14 years of experience. All the graders and the adjudicators were certified after completing an online UK DR grading course (Diabetic retinopathy grading course awarded by Gloucestershire Retinal Education Group. Gloucestershire Hospital NHS Foundation Trust, UK) and successfully passing the final evaluation exam.

**Table 1:** Scottish diabetic retinopathy grading scheme [16]

Grade	Description
*Retinopathy (R)*	
R0	No visible retinopathy
R1 (mild)	The presence of any of following: Dot or blot hemorrhage Microaneurysms Hard exudate Cotton wool spots Superficial flame shape hemorrhage
R2 (observable)	4 or more blot hemorrhage in one hemi-field only
R3 (referable)	Any of the following features: 4 or more blot hemorrhage in both hemi-field venous beading IRMA
R4 (proliferative)	Any of the following features: Active new vessels Vitreous hemorrhage
R5 (inadequate)	Not adequately visualized Retina not sufficiently visible for assessments
*Maculopathy (M)*	
M0	No signs of maculopathy
M1 (observable)	Lesions as specified bellow within a radius > 1 but ≤ 2 disk diameters the center of the fovea Any hard exudates
M2 (referable)	Lesions as specified bellow within a radius of 1 but ≤ 1 disk diameters the center of the fovea Any blot hemorrhage Any hard exudates
IRMA—intra-retinal microvascular abnormality	

For all the gradable photographs, diabetic maculopathy would be graded as either referable (M2) or non-referable (M0 or M1).

A quality assurance check was conducted by re-grading 5% of the non-conflicted images by one of the two consultant adjudicators (AE or IA). Each grader received individual feedback on his or her performance. The overall agreement between the non-conflicted grade and the consultant adjudicators was 79%.

After grading the fundus photographs, a report was generated by the software system and sent to the patient by e-mail or SMS. The report included the grade of DR, the presence of suspected DMO for each eye, the recommended time for the next fundus screening, and the need for hospital referral. Referable cases included DR grade *R*3 or worse, diabetic maculopathy grade M2 or non-gradable photographs in one or both eyes.

### The grading software

#### Pilot screening system

The study aimed to provide a pilot screening system. To achieve this goal, pilot software was designed and developed. The screening system is accessible through the web. The system defines multiple roles and privileges to control and monitor the process of gathering the patients' data and grading the images.

#### Data collection software

The data collection software is designed to allow the nurse to gather required patient and exam information. Patient information includes national ID, place of birth, address, date of birth, and scanned informed consent. The exam information includes the diabetes type, current blood sugar level, latest known HbaA1c, and fovea-centered fundus images for two eyes of the patient. All the data are stored locally and synchronized into a centralized database on local cloud storage inside Egypt.

#### Data validation software

The data validation software is designed to allow the graders to access and evaluate the fundus images that were collected previously. Every grader has access to a pooled list of ready-to-grade images. The software aggregates the grades from the graders. In the case of a conflict, the adjudicator will be able to access a pooled list of ready-to-adjudicate images. The final decision is then returned to the system.

#### Administrator portal

The portal allows the administrator of the pilot to generate statistics on the data collection and grading activity. Reporting and patient notification are done through the administrator portal as long as the patient's exam is fully graded.

### Statistical analysis

Python 3.7.5 was used to perform the statistical analysis using the statsmodels 0.13.2 and scipy 1.7.1 libraries. Frequencies (number of cases) and percentages were used to statistically describe the data when appropriate. The Pearson’s *χ*2-test, *t*-test, and ANOVA tests were used to compare the categorical, the means of the continuous variables of two groups, and those of more than two groups, respectively.

Univariate logistic regression was used to assess the presence or absence of DR with each factor individually and to build the final multivariate regression model. *p* values < 0.05 were considered statistically significant.

## Results

### Demographic details

All the included patients were of Middle Eastern ethnicity. Out of the total of 11,033 screened diabetic patients, 222 had ungradable images in both eyes and were excluded from the grading analysis. A final number of 10,811 patients who had a gradable quality in at least one eye were included for statistical analysis.

The mean age of the patients was 55 ± 12.63 years. Male patients represented 38.4% of the patients (4147/10811). The mean duration of diabetes was 11.26 ± 8.44 years. Most of the included patients (8249 patients, representing 76.3% of the included patients) suffered from type 2 DM, while 2562 patients (23.7%) suffered from type 1 DM.

We divided the included patients into three groups; Group 1 included 2826 patients (26.1%) who were recruited from the general ophthalmology clinics. Patients recruited from the diabetes internal medicine clinics represented Group 2, including 4045 patients (37.4%). Group 3 was the largest and represented the diabetic patients recruited from the local communities. It included 4045 patients (37.4%).

### Diabetic retinopathy

The DR grades prevalence was calculated based on the worst affected eye. The prevalence of grades *R*0, *R*1, *R*2, *R*3 and *R*4 for all patients was 67.5%, 15.5%, 6%, 4.8%, and 6.2%, respectively.

The prevalence of No DR (*R*0) was significantly higher in patients with type 2 than type 1 DM (70.39% vs 58.23%, *p* < 0.001). Grade *R*4 was significantly higher in patients with type 1 than type 2 DM (10.46% vs 4.83%, *p* < 0.001). (Table 2).

**Table 2:** Patient demographics and prevalence of retinopathy and maculopathy according to worse eye in patients with type 1 and type 2 diabetes

	All, *n* = 10,811	Type 1, *n* = 2562 (23.7%)	Type 2, *n* = 8249 (76.3%)	*p* value	No retinopathy, *n* = 7299	Any retinopathy, *n* = 3512	*p* value
Age, mean (SD)	55 (12.63)	50 (14.27)	57 (11.63)	< 0.001	54 (13.18)	57 (11.15)	< 0.001
Gender n (%), males	4147 (38.35)	834 (32.55)	3313 (40.16)	< 0.001	2780 (38.09)	1367 (38.92)	0.414
Random blood glucose, mean (SD)	199.36 (94.81)	219.97 (105.05)	191.52 (89.38)	< 0.001	193.22 (93.53)	210.78 (96.13)	< 0.001
Duration of diabetes (years), mean (SD)	11.26 (8.44)	13.34 (9.03)	10.62 (8.14)	< 0.001	8.8 (7.32)	16.35 (8.33)	< 0.001
Diabetes type *n* (%)							
Type 1	2562 (23.7)	NA	NA	NA	1492 (58.23)	1070 (41.76)	< 0.001
Type 2	8249 (76.3)				5807 (70.39)	2442 (29.6)	
Retinopathy Stage *n* (%)							
None	7299 (67.51)	1492 (58.23)	5807 (70.39)	< 0.001	7299 (100)	0 (0)	NA
R1	1675 (15.5)	439 (17.13)	1236 (14.98)		0(0)	1675 (47.69)	
R2	648 (6)	202 (7.9)	446 (5.4)		0 (0)	648 (18.45)	
R3	522 (4.8)	161 (6.3)	361 (4.37)		0 (0)	522 (14.86)	
R4	667 (6.16)	268 (10.46)	399 (4.83)		0(0)	667 (19)	
Maculopathy Stage *n* (%)							
No referable maculopathy (M0, M1)	8654 (80.05)	1859	8654 (80.05)	< 0.001	7299 (100)	1355 (38.58)	NA
Referable maculopathy (M2)	(72.56)	6795	(72.56)		0 (0)	2157 (61.42)	

The prevalence of no DR (*R*0) was highest in Group 3 (78.7%) and lowest in Group 1 (53.6%). Group 2 had an intermediate prevalence (70.9%). This difference was statistically significant (*p* < 0.001).

On the other hand, the patients from Group 1 had the highest prevalence of proliferative DR (grade *R*4) (11.8%). Patients from Group 2 (5%) had an intermediate prevalence while those from Group 3 had the lowest (1.5%). The difference between the groups was statistically significant (*p* < 0.001). (Table 3).

**Table 3:** Patient demographics and prevalence of DR and maculopathy for diabetic patients included from the general ophthalmology clinics, diabetic internal medicine clinics and community

	General ophthalmology clinics (Group 1) *n* = 3940 (36.5%)	Diabetic internal medicine clinics (Group 2) *n* = 2826 (26.1%)	Community (Group 3) *n* = 4045 (37.4%)	*p* value
Age, mean (SD)	58 (11)	54 (12)	53 (14)	< 0.001
Gender *n* (%), males	1480 (37.56)	1053 (37.26)	1614 (40)	0.038
Random blood glucose, mean (SD)	198.62 (91.55)	203.81 (98.06)	195.12 (95.06)	0.005
Duration of diabetes (years), mean (SD)	13.77 (8.79)	11.29 (8.14)	8.77 (7.52)	< 0.001
Diabetes type *n* (%)				
Type 1	1091 (27.69)	767 (27.14)	704 (17.4)	< 0.001
Type 2	2849 (72.3)	2059 (72.85)	3341 (82.59)	
Unspecified	0 (0)	0 (0)	0 (0)	
Retinopathy stage *n* (%)				
None	2112 (53.6)	2003 (70.87)	3184 (78.71)	< 0.001
R1	675 (17.13)	455 (16.1)	545 (13.47)	
R2	376 (9.54)	131 (4.63)	141 (3.48)	
R3	311 (7.89)	95 (3.36)	116 (2.86)	
R4	466 (11.83)	142 (5.02)	59 (1.45)	
Maculopathy stage *n* (%)				
No referable maculopathy (M0, M1)	2702 (68.58)	2376 (84.08)	3576 (88.41)	< 0.001
Referable maculopathy (M2)	1238 (31.42)	450 (15.92)	469 (11.59)	

### Diabetic maculopathy

The overall prevalence of referable maculopathy (M2) was 19.95%. The prevalence of grade M2 was significantly higher in patients with type 1 DM (27.44%) than in patients with type 2 DM (17.63%) (*p* < 0.001) (Table 2). The M2 prevalence was highest in patients from Group 1 (31.42%), intermediate in patients from Group 2 (15.92%) and it was lowest in patients from Group 3 (11.59%). The difference was statistically significant (*p* < 0.001). (Table 3).

In univariate analysis, older age, higher random blood glucose, and longer duration of DM were associated with a higher risk of both DR and referable diabetic maculopathy. This association with both DR and referable diabetic maculopathy was maintained in multivariate analysis for the high random blood glucose level and the longer duration of DM, but not for the older age. In either univariant or multivariant analysis, higher HbA1c levels were not associated with a higher risk of either DR or referable maculopathy (Tables 4, 5).

**Table 4:** Univariate and multivariate logistic regression showing the risk factors associated with the development of diabetic retinopathy in diabetic patients

	Univariate regression			Multivariate regression		
	Odds ratio	95% CI	*p* value	Odds ratio	95% CI	*p* value
Age	1.019	1.016–1.022	< 0.001	1	0.993–1.007	0.998
Gender: male vs. female	1.036	0.954–1.125	0.402	1.112	0.917–1.35	0.281
HbA1c	1	0.999–1.001	0.822	1	0.999–1.001	0.9
Random blood glucose	1.002	1.001–1.002	< 0.001	1.002	1.001–1.003	0.001
Duration of diabetes (years)	1.125	1.119–1.132	< 0.001	1.112	1.097–1.126	< 0.001

**Table 5:** Univariate and multivariate logistic regression showing the risk factors associated with the development of diabetic maculopathy in diabetic patients

	Univariate regression			Multivariate regression		
	Odds ratio	95% CI	*p* value	Odds ratio	95% CI	*p* value
Age	1.017	1.013–1.021	< 0.001	1.002	0.993–1.01	0.710
Gender: male vs. female	1.024	0.93–1.127	0.635	1.071	0.857–1.339	0.545
HbA1c	1	0.999–1.001	0.985	1	0.998–1.001	0.747
Random blood glucose	1.002	1.002–1.003	< 0.001	1.002	1.001–1.003	0.003
Duration of diabetes (years)	1.1	1.094–1.106	< 0.001	1.094	1.08–1.108	< 0.001

## Discussion

Diabetes mellitus is a growing health problem in different parts of the world. Egypt is not an exception. Herman et al. [17] estimated in a paper published in 1995 that by 2025 nearly 13% of the Egyptians (about 9 million people over 20 years of age) will suffer DM.

The current study reports the results of the DR screening program in Alexandria and the North-West Delta in Lower Egypt. Alexandria is the second-largest city in the country after the capital (Cairo), and the study region has a high-density population. To the best of our knowledge, the current study is the largest one conducted in Egypt, the Middle East, and Africa so far in terms of the included population There is a high variability in the reported prevalence of DR both in Africa (20- > 50%) and in the Middle East Area (19- > 48%) (Table 6). This may be attributed to the variability in the ethnic background or the variation in the examination tools, screening and/or grading protocols. Most of the reported studies included a relatively small number of diabetics, and many of them were not population-based.

**Table 6:** Summary of the previously published prevalence of DR in Africa, the Middle East Area and Egypt

Country	Author Date (Ref)	Study period	Study type	Study location	Population					Grading scheme	Diabetic retinopathy (%)	DR grade						Maculopathy	
					n (Total)	T1 DM	T2 DM	Mean Age (Range)	Sex M (%)		Any DR	Mild BDR	Mod PPDR		Severe PPDR		PDR	DME	CSME
Africa																			
Ethiopia	Alemu 2015 [18]	NR	X-section	Prim Care	511	Yes	No	34.6	62.1	Diabetic Retinopathy Study guidelines	8.6 R = 5.0 U = 16.1	2.2 0.6 5.4	4.9 3.8 7.1		0.4 0 1.2		1.2 0.6 2.4	6.3 3.8 11.3	
Tanzania	Cleland 2016 [19]	2010–2014	X-section	Comm	3187	NR	NR	60.8	39.7	English and Wales National Screening Committee	27.9	19.1	6.0				2.9	16.1	
S Africa	Webb 2016 [19]	2010–2012	Cluster R Trial	Prim Care	497	18	334 + unspec	57.8 (20–90)	31.5	Scottish	24.9	19.5					5.5	20.8	9.0
S Africa	Cairncross 2017 [19]	2014	Prospective	Hospital	185	Yes	Yes	57 (30–88)	27.5	Scottish	20	10.2	3.6		2.5		3.1	12.7	
Zambia	Lewis 2018 [20]	2012	X-section	Comm	2153	208	921 + unspec	56	55	NHS	All 51.7 T1DM = 61.5 T2DM = 52.2	26 26 28	20 29 20				6 7 5	32 54 41	
Middle East																			
Saudi Arabia	Al-Rubeaan 2015 [21]	NR	X-section	Diabetic registry	50 464	No	Yes	59.7	56	NR	19.7	9.1					10.6	5.7	
Saudi Arabia	Al Ghamdi 2012 [22]	NR	X-section	Comm	3052	NR	NR	63.3	54	Scottish	34.5				17.5 (STDR) sight threatening			20.3	
Bahrain	Al Alawi 2012 [22]	2003–2009	X-section	Primary, secondary and shared care	17,490	4	89 + 7 diet controlled	53 (24–84)	51	NR	20.4	15.3	2.3		0.7		1.2	5.2	
Jordan	Rabiu 2015 [22]	NR	X-section	Comm	3780	NR	NR	≥ 50	50.4	Scottish	48.4	20.8	11.8		6.4		8.3	28.5	11
Iran	Heydari 2012 [22]	2008–2010	X-section	Hospital	*1022*	NR	NR	55.7 (5–87)	40.2	ETDRS	*23.6*								
Saudi Arabia	*Hajar 2015* [23]	2011–2012	X-section	Comm	3659	NR	NR	63.3	51.2	Scottish	27.8	18	5.3		3.5		1.1	13.1	5.3
UAE	*Al-Maskari 2007* [24]	2003–2004	X-section	Prim care	513	68	431	53.3	52	Watkins et al (2003) standards	19	13.8					3.8 + 1.7 advanced diabetic eye disease		
Kuwait	Al-Adsani 2007 [25]	2000–2005	X-section	Hospital	165	NR	165	48.99	33.9	NR	40	21.2		7.9			3	10.3	
Egypt																			
	Macky 2011 [26]	2007–2008	X-section	Hospital	1,325	354	971	49	28.5	the International Clinical Diabetic Retinopathy Disease Severity Scale	20.5 23 T1 DM 21 (T2 DM)	6.7 (No DME) 11.5 (With DME)					2.3		
	AlSawahli 2021 [27]	2019	X-section	Comm	729	NR	NR	≥ 50	61.9	Scottish	13.7 (any DR) 17.9 (DR)	7		3.2		2.6	1	9.7	4.5
	Herman 1998^28^	1998	X-section	Comm	1451	NR	NR	≥ 20	NR	NR	42								

There are a few reports about the prevalence of DR in Egypt. None of them studied the prevalence of DR in Alexandria and the nearby governorates [26–28]. (Table 6).

In the current study, 23.7% of the patients suffered from type 1 DM and 76.3% suffered from type 2 DM. This is like the reports from the other countries in the Middle East [3, 8–10, 12–14, 22, 23, 26, 28–35]. On the other hand, it is reported that most of the diabetics in sub-Saharan Africa (> 90%) suffer from type 2 DM and that type 1 occurs predominantly in patients of European ancestry [36, 37].

The mean age onset of DM in patients with type 1 DM in the current study was higher than average. This could be explained with the fact that our reach out campaigns were done in the morning time. At that time, most of the younger patients were at their schools, universities or work. Most of the enrolled patients attending the hospital or reached out in the community were of older age.

Currently, there is no existing regular screening program for DR in Egypt. The current study was a good initiative to build a database of diabetic patients, and hopefully, it will help in reducing the visual impairment due to DR.

We recruited the patients in the current study from 3 different sources; the general ophthalmology clinics in which the patients are not specifically coming for the management of the DR, the diabetic internal medicine clinics for which the patients are usually coming for the management of poor diabetic control, and from the reach out campaigns in the local communities, which we believe will give a better picture of the magnitude of the problem, as many diabetic patients may suffer DR but not seek medical advice early if they are still asymptomatic.

We found that patients with type 1 DM had a significantly higher level of sight-threatening complications than patients with type 2 DM, like proliferative DR (Grade *R*4) (10.46 vs 4.83%, *p* < 0.001) and referable maculopathy (Grade M2) (27.44 vs 17.63%, *p* < 0.001). Similar results were reported from other studies [20, 26].

Patients attending the ophthalmology clinics in the present study had the highest prevalence of proliferative DR (*R*4) (11.83%) and referable maculopathy (M2) (32.92%). The prevalence was lowest in the patients from the community (only 1.45% for *R*4 and 12.46% for M2) and it was intermediate for the patients attending the diabetic internal medicine clinics (5.02% for R4 and 17.45% for M2). The prevalence variation draws attention to the importance of the primary prevention of DR by maintaining good metabolic control and the secondary prevention through the early DR screening in the community before the progression of the sight-threatening complications. Alemu et al. reported a higher prevalence of DR and DME in urban areas than in rural areas in Ethiopia [18]. In the current study, the reach out campaigns recruited diabetic patients from both urban and rural areas.

In the patients recruited from the local communities in the current study, we found that the prevalence of any DR (21.29%) and that of grade *R*4 (1.45%) is similar to the results of AlSawahli et al. who conducted a population-based study of the prevalence of DR using the same grading scheme in 2019 on patients from Sohag governorate in Upper Egypt. They reported the prevalence of any DR and of grade *R*4 to be 17.9% and 1%, respectively. On the other hand, we found that 12.46% of the patients in this group had grade M2 which is higher than the prevalence reported by AlSawahli et al. (4.5%) [27].

In the current study, we found a slightly higher prevalence of any DR and of grade *R*4 in the patients recruited from the diabetic internal medicine clinics (29.13% and 5.02%, respectively) than that reported by Macky et al. who reported a prevalence of 20.5% for any DR and of 2.3% for PDR in a hospital-based sample carried out in the Cairo metropolitan area on patients attending the diabetic endocrinology clinics between 2007–2008. This difference can be attributed to the possible rise in the prevalence of DR over the years or due to differences in the sampling methods and the DR grading scheme used in each study [26].

In the present study, we did not find a significant difference in the prevalence of the DR between males and females. Macky et al. [26] reported a higher DR prevalence in females (22%) than in males 17%. AlSawahli et al. [27] reported the same finding (DR prevalence was 18.9% in females and 17.1% in males).

Most of the included patients in the current study were females (> 60%). This is similar to other epidemiological studies on diabetic patients in Egypt and other countries from the same region [28–30, 33, 38–40].

In the present study, patients with higher random blood glucose and longer duration of DM were at a higher risk of both DR and referable diabetic maculopathy on both univariate and multivariate analysis [26, 27]. Older age was a significant risk factor on univariate but not on multivariant analysis. A similar weak association was reported in a previous study [26]. HbA1c was not associated with a higher risk of either DR nor referable maculopathy on either univariant or multivariate analysis. This surprising finding was previously reported [26, 33] and it may be attributed to the fact that HbA1c reflects glycemic control only over the last 3 months but not before that.

In the present study, we found a significantly higher prevalence of diabetic retinopathy in patients with type 1 DM in comparison with those with type 2 DM (41.77% vs 29.61%). Similar results were found for both grade *R*4 (10.46% vs 4.83%) and grade M2 (27.44% vs 17.63%). This is different from the results reported previously by Macky et al. [26] which could be due to the difference in the type of the patient’s sources.

Only a few previous reports discussed the prevalence of DR in diabetics from Egypt. All of them studied smaller samples (729–1451 patients). None of them included patients from different sources (community, ophthalmology clinics and internal medicine diabetic clinics).

The current study showed a higher prevalence of DR among Egyptian diabetic patients. Which demonstrates that the DR is a growing general health problem that needs a lot of efforts to control.

In addition, the current study showed a significantly lower prevalence level of DR, higher grade DR and referable maculopathy in patients reached out in the community than in patients coming to the hospitals. This clearly shows the importance of the DR screening program for the early detection of the condition before the occurrence of any visually disabling complications. This information was added to the discussion section.

In the current study, we included only patients with confirmed diagnoses of DM. The true prevalence of DR may actually be higher than reported as some of the population might suffer from undiagnosed DM and some known diabetic patients might not be motivated to participate in the study.

The current study has several limitations. Firstly, the lack of a registry of the diabetic patients in Egypt made reaching out for diabetic patients more challenging. In the current study, we depended on self-reporting of diabetic patients. In addition, we could not get accurate data about the medical history of the patients (e.g., duration, control and type of DM). Most of the reach out campaigns in the community were done in the morning time. This probably was a possible cause to miss younger diabetics attending the school, university or work.

We believe that our study highlights the importance of primary prevention of the diabetic retinopathy through adequate control of the potential risk factors. In addition, the present study demonstrates the importance of reaching out to the diabetic patients in the local communities through an efficient screening program to detect and treat cases early and in a cost-effective way.
